# Autoimmunity as a Candidate for the Etiopathogenesis of Meniere's Disease: Detection of Autoimmune Reactions and Diagnostic Biomarker Candidate

**DOI:** 10.1371/journal.pone.0111039

**Published:** 2014-10-17

**Authors:** Sung Huhn Kim, Jin Young Kim, Hyun Jin Lee, Mia Gi, Bo Gyung Kim, Jae Young Choi

**Affiliations:** 1 Department of Otorhinolaryngology, Yonsei University College of Medicine, Seoul, Korea; 2 The Airway Mucus Institute, Yonsei University College of Medicine, Seoul, Korea; 3 Research Center for Human Natural Defense System, Yonsei University College of Medicine, Seoul, Korea; University of South Florida, United States of America

## Abstract

Meniere's disease is an inner ear disorder that can manifest as fluctuating vertigo, sensorineural hearing loss, tinnitus, and aural fullness. However, the pathologic mechanism of Meniere's disease is still unclear. In this study, we evaluated autoimmunity as a potential cause of Meniere's disease. In addition we tried to find useful biomarker candidates for diagnosis. We investigated the protein composition of human inner ear fluid using liquid column mass spectrometry, the autoimmune reaction between circulating autoantibodies in patient serum and multiple antigens using the Protoarray system, the immune reaction between patient serum and mouse inner ear tissues using western blot analysis. Nine proteins, including immunoglobulin and its variants and interferon regulatory factor 7, were found only in the inner ear fluid of patients with Meniere's disease. Enhanced immune reactions with 18 candidate antigens were detected in patients with Meniere's disease in Protoarray analysis; levels of 8 of these antigens were more than 10-fold higher in patients than in controls. Antigen-antibody reactions between mouse inner ear proteins with molecular weights of 23–48 kDa and 63–75 kDa and patient sera were detected in 8 patients. These findings suggest that autoimmunity could be one of the pathologic mechanisms behind Meniere's disease. Multiple autoantibodies and antigens may be involved in the autoimmune reaction. Specific antigens that caused immune reactions with patient's serum in Protoarray analysis can be candidates for the diagnostic biomarkers of Meniere's disease.

## Introduction

**Figure 1:**
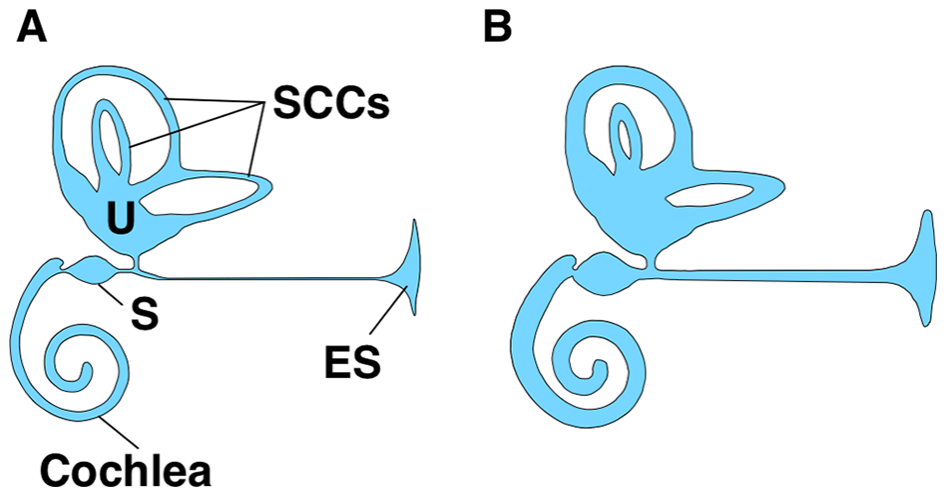
Schematic drawing of the inner ear and endolymphatic hydrops as a mechanism for Meniere's disease. The inner ear consists of the cochlea, vestibule, and endolymphatic sac (ES). The utricle (U), saccule (S), and semicircular canals (SCCs) form the vestibule. A. Normal inner ear structure. B. Endolymphatic hydrops in patients with Meniere's disease.

In 1861, Prosper Meniere first described Meniere's disease as an inner ear disorder that manifests as fluctuating vertigo, sensorineural hearing loss, tinnitus, and aural fullness. The prevalence of Meniere's disease is 3.5–513 per 100,000, which is higher than the prevalence of systemic lupus erythematosus (SLE) and multiple sclerosis [1]. The unpredictable nature of Meniere's disease has a serious effect on patients' daily life. During active episodes, the quality of life score of patients with Meniere's disease is thought to be lower than that of AIDS patients treated with AZT, that of patients with severe chronic obstructive pulmonary disease, and that of non-institutionalized patients with Alzheimer's disease [2]. The main pathologic site is thought to be the inner ear, which consists of the cochlea, vestibule, and endolymphatic sac. A characteristic finding of Meniere's disease is the dilatation of the endolymphatic compartment of the inner ear caused by an increase in endolymph (endolymphatic hydrops, Fig. 1) [3]. The proposed etiologies of endolymphatic hydrops are autoimmune, allergic, genetic, traumatic, and infectious (viral) [4]–[9]. These finally result in endolymphatic hydrops by deteriorating ion homeostasis and fluid volume regulation in the inner ear [3]. However, the exact pathologic mechanism underlying endolymphatic hydrops is still unknown.

Certain findings have provided evidence that autoimmunity may underlie the pathology of Meniere's disease. The prevalence of systemic autoimmune diseases such as rheumatoid arthritis, ankylosing spondylitis, and SLE in patients with Meniere's disease is 3- to 8-fold higher than in the general population [10]. In addition, autoantibodies such as the anti-heat-shock protein 70, anti-68 kD inner ear protein antibody, anti-myelin peroxidase zero antibody, and anti-thyroid peroxidase antibody have been detected in the serum of patients with Meniere's disease [11]–[14]. However, these autoantibodies were not found in all of the patients.

Previous studies tended to investigate only a select few target proteins instead of conducting mass screening; in addition, many of these studies used western blot analyses to detect antigen-antibody reactions between patient serum and animal inner ear tissues, which can demonstrate the existence of an antigen-antibody reaction but provides no information on the identity of the autoantibody. Few studies demonstrated increased proteins in the serum of Meniere's disease patients that were reported to be related with inflammatory reaction or inner ear disorders by proteomics technique [15]. But, there was no evidence if these materials existed in the inner ear fluid of Meniere's disease patients. Studies using human inner ear tissue are rare, and no studies have investigated autoimmunity using human inner ear fluid. To overcome the limitations of previous studies and to understand the autoimmune pathologic mechanisms underlying Meniere's disease, mass screening-based studies of autoimmune reactions using human inner ear fluid and sera of patients should be conducted.

In this study, we tried to provide evidence for the involvement of autoimmunity in Meniere's disease and identify the candidate antigens that react with autoantibodies, which can suggest diagnostic biomarker candidates for Meniere's disease. Several studies were performed. First, the protein composition of inner ear fluid from control patients and patients with Meniere's disease was compared using proteomic analysis. Second, candidate autoantigens that reacted with circulating autoantibodies in patient serum were investigated using protein array (Protoarray, Invitrogen, Life Technologies, Grand Island, NY). Third, western blots using patient serum and mouse inner ear tissues were performed to investigate whether the circulating autoantibodies reacted with inner ear tissue. The results of this study can provide the basic information for the development of diagnostic biomarkers as well as the understanding of pathologic mechanisms of Meniere's disease.

## Methods

### Selection of patients and controls

Thirteen patients diagnosed with Meniere's disease according to the criteria of the American Academy of Otolaryngology Head and Neck Surgery (1995) [16] were enrolled in the patient group. Samples of inner ear fluid (endolymphatic sac luminal fluid) were taken from 3 patients undergoing endolymphatic sac surgery to treat their intractable disease, and peripheral blood was sampled from the other 10 patients. Three patients with acoustic tumors and 10 patients with simple tympanic membrane perforation who planned to undergo myringoplasty were enrolled as controls. Endolymphatic sac luminal fluid was sampled from the 3 controls during the acoustic tumor surgery via the translabyrinthine approach, and peripheral blood was sampled in the other 10 controls. The controls with acoustic tumors had severe sensorineural hearing loss but no history of sudden vertigo. The controls had no history of sensorineural hearing loss or vertigo, and their audiograms showed mild conductive hearing loss. None of the patients or controls had a history of systemic disease, and all of their laboratory parameters, including the electrocardiogram, chest radiography, blood cell counts (red blood cells, white blood cells, and platelets), liver and kidney function tests, and urinalysis, were normal. The gender distribution and mean age of the patients and controls were not significantly different (p>0.05 for the chi-square test and t-test, Table 1).

**Table 1:** Demographics of patients and controls.

	Inner ear fluid analysis	Peripheral blood analysis
	Male to female ratio	
Controls	1:2	7:3
Patients	1:2	5:5
	Mean age in years (mean ± SD)	
Controls	47.3 ± 5.5	43.1 ± 7.8
Patients	39.0 ± 22.5	49.6 ± 7.0

### Sampling of inner ear fluid and sera

Three patients with Meniere's disease underwent endolymphatic sac surgery for their intractable disease and the 3 controls with acoustic tumors underwent tumor removal via the translabyrinthine approach. To obtain inner ear fluid, endolymphatic sac luminal fluid was sampled during surgery. In each surgical procedure, the endolymphatic sac should be fully exposed. Because the amount of luminal fluid was very small (<4 µl), we infused 200 µl of normal saline into the endolymphatic sac and aspirated the diluted luminal fluid from the endolymphatic sac, as previously described [17]. The fluid samples were immediately stored at −80°C until analysis.

Blood was sampled from the other 10 participants in each group for serum protein analysis. The blood was stored in a tube containing ethylenediaminetetraacetic acid. The plasma was immediately separated and stored at −80°C until analysis.

### One-dimensional electrophoresis (1-DE) of endolymphatic sac luminal fluid

1-DE was performed to compare the protein composition of the fluid of patients with Meniere's disease and that of controls. Thirty micrograms of protein from the diluted luminal fluid was used for 1-DE for each sample. The samples were lyophilized and dissolved in 15 µl of distilled water. Samples were subjected to sodium dodecyl sulfate gel electrophoresis on an 8–16% Tris/Glycine gel and stained with Coomassie Brilliant Blue.

### Identification of endolymphatic sac luminal fluid proteins by liquid column mass spectrometry (LC-MS/MS)

The entire 1-DE gel lane was cut into 8 pieces according to molecular weight for digestion. After reduction with dithiothretol and alkylation with iodoacetamide, each piece of gel was treated with trypsin for *in situ* digestion. It was then washed with 10 mM ammonium bicarbonate and 50% acetonitrile, swollen in digestion buffer containing 50 mM ammonium bicarbonate, 5 mM CaCl_2_, and 1 µg of trypsin. Next, it was incubated at 37°C for 12 h. Peptides were recovered over the course of 2 extraction cycles with 50 mM ammonium bicarbonate and 100% acetonitrile. The resulting peptide extracts were pooled, lyophilized, and stored at −20°C.

Nano LC-MS/MS analysis was performed on an Agilent 1100 Series nano-LC and linear trap quadrupole (LTQ)-mass spectrometer (Thermo Electron, San Jose, CA). The capillary column used for LC-MS/MS analysis (150 mm ×0.075 mm) was obtained from Proxecon (Odense M, Denmark) and slurry-packed in-house with 5 µg, 100 Å pore size Magic C18 stationary phase (Michrom Bioresources, Auburn, CA). The mobile phase A for LC separation was 0.1% formic acid in deionized water and the mobile phase B was 0.1% formic acid in acetonitrile. Chromatography was performed using a linear gradient from 5% B to 35% B over 100 min, from 40% B to 60% B over 10 min, and from 60% B to 80% B over 20 min. The flow rate was maintained at 300 nl/min after splitting. Mass spectra were acquired using data-dependent acquisition with full mass scan (400–1800 m/z) followed by MS/MS scans. Each acquired MS/MS scan represented the average of one microscan on the LTQ. The temperature of the ion transfer tube was controlled at 200°C and the spray was 1.5–2.0 kV. The normalized collision energy was set at 35% for MS/MS.

The MASCOT and SEQUEST (BioWorks software version 3.2, Thermo Electron) search engines were used to search the UniProt human protein databases (release 14.8; 82728 sequences) for the tandem mass spectra. Mass tolerances of 1.2 Da and 0.6 Da were used for precursor and fragment ions, respectively. The search included variable modification of oxidation on methionine and carbamidomethyl of cysteine. PeptideProphet and ProteinProphet were used to estimate the false discovery rate (FDR) for any minimum probability used as a cut-off for MASCOT and SEQUEST search results.

### Protoarray analysis of serum samples (Immune Response Biomarker Profiling)

To investigate the presence of autoantibodies and their target antigens, Protoarray (Human Protein Microarray v5.0 containing 9400 human proteins, Invitrogen) analysis was performed with sera from patients and controls according to the manufacturer's protocol. Briefly, after blocking array slides with blocking buffer for 1 h, we washed the slides with washing buffer for 5 min, and 5 ml of diluted serum (1∶500) was placed on the slides. The slides were incubated for 90 min and washed 4 times for 5 min with washing buffer. After washing, Alexa Fluor 647 (final concentration of 1 µg/ml) was added on the slide, and the slides were incubated for 90 min. The antibody was aspirated, and the slides were washed for 5 min 4 times. These steps were performed at 4°C. The slides were dried immediately by centrifugation at 200×g for 1 min and stored in a slide box to protect them from light until scanning was performed. The dried arrays were scanned using a GenePix 4000B microarray scanner (Molecular Devices, Sunnyvale, CA). Genepix Pro microarray data acquisition software was used to align the scanned image with the template and to acquire the pixel intensity data for each spot on the array. The reported pixel intensity was calculated as the average of duplicate signals obtained after subtracting the background signal. Protoarray Prospector software (Life Technologies) was used to analyze the data, perform background subtraction, and normalize the signals. The normalized signal intensities obtained for controls and patients were compared with a t-test, and differences were considered significant at p<0.05.

### Western blotting

To investigate whether antibodies in the patient serum reacted with inner ear tissue, western blots using patient serum and mouse inner ear tissue were performed. We used mouse inner ear tissue for ethical reasons and because it would have been technically difficult to harvest the entire human cochlea and vestibule. Eight-week-old male C57BL/6 mice were used. The entire inner ear was separated from the temporal bone of the mouse. The cochlea and vestibule were separated, and the membranous labyrinth of the cochlea and vestibule were carefully dissected. Each membranous labyrinth was lysed with 2× sample buffer (250 mM Tris-HCl [pH 6.5], 2% sodium dodecyl sulfate (SDS), 1% DTT, 0.02% bromophenol blue, and 10% glycerol). Protein levels were quantified by comparing the absorbance of the lysate with that of serially diluted bovine serum albumin (0, 0.2, 0.4, 0.6, 0.8, and 1 mg/ml) in the VersaMax ELISA plate reader (Molecular Devices). Samples were heated for 5 min at 95°C. Equal total amounts of protein were prepared for each gel lane. A colored marker mixture was used to estimate the molecular weights of the bands. Proteins were separated using 10% SDS-polyacrylamide gel at 125 V for 4 h with a running buffer (25 mM Tris-Base, 192 mM glycine and 0.1% SDS) and transferred to polyvinylidene difluoride membranes (Millipore, Bedford, MA) using a semi-dry transfer cell (Bio-Rad, Hercules, CA) for 2 h at 200 mA and a transfer buffer (10 mM Tris-HCl [pH 8.0], 150 mM NaCl, 0.05% Tween 20). The membranes were blocked with 5% skim milk in Tris-buffered saline (TBS; 50 mM Tris-HCl [pH 7.5] and 150 mM NaCl) for 2 h at room temperature. The blot was incubated overnight with patient serum diluted at 1∶200 in 0.5% Tween-20 in TBS. The blot was washed with TTBS, incubated with a secondary anti-human antibody (Cell Signaling Technology, Danvers, MA) in TTBS for 45 min at room temperature, and visualized by enhanced chemiluminescence (Amersham Biosciences, Champaign, IL).

### Ethics statement

This study was approved by the institutional review board of Severance Hospital (approval number 4-2011-0871 and 4-2013-0483), and written informed consent was obtained from all of the participants. Institutional Animal Care and Use Committee of Yonsei University College of Medicine approved this study and all mice were treated in accordance with the guidelines for the Care and Use of Laboratory Animals of Yonsei University College of Medicine (approval number 2011-0084).

## Results

### Difference in the protein composition of the ES luminal fluid of controls and patients

**Figure 2:**
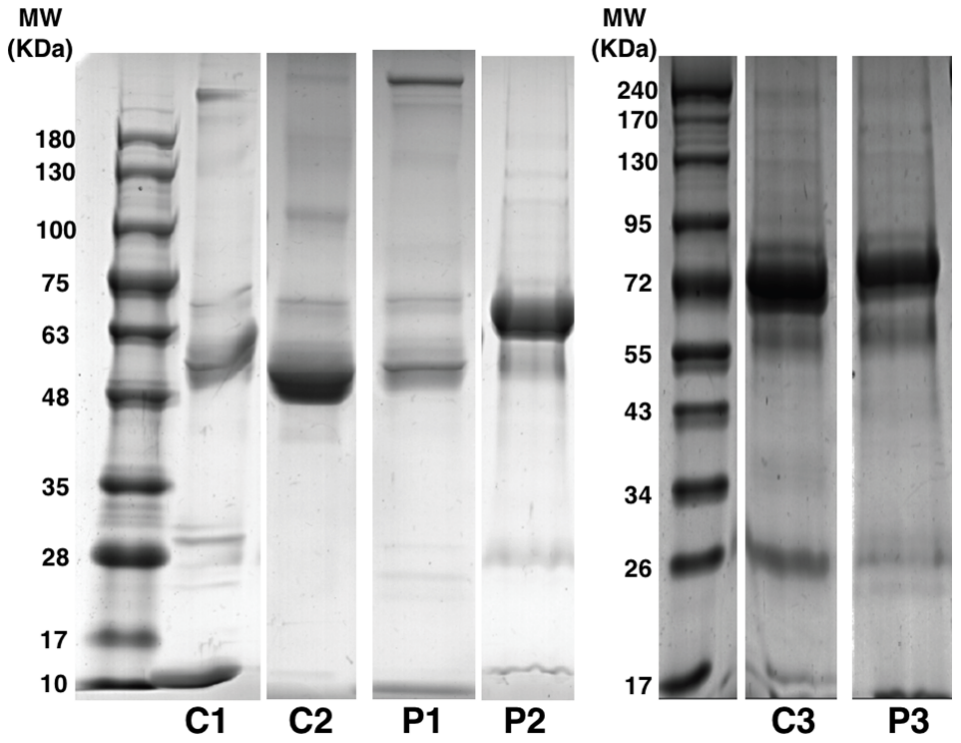
1-DE of the proteins in the endolymphatic sac luminal fluid of each control and patient. The distribution of bands in each individual varied. C, control; P, patient.

The density and distribution of protein bands in the 1-DE of ES luminal fluid from controls and patients with Meniere's disease varied (Fig. 2); the density and molecular weight of bands from the patients and controls were identical in some cases and different in others. For example, the bands located between 63 and 75 kDa associated with control 1 and 2 (C1 and C2) and patient 1 (P1) appeared similar, but the bands associated with control 3 (C3) and patient 2 and 3 (P2 and P3) appeared denser. The bands located between 17 and 35 kDa and between 75 and 180 kDa also varied. Consequently, the distribution and density of protein bands differed for each individual, and it was difficult to identify disease-specific protein bands.

To identify differences in the protein composition of the ES luminal fluid of controls and patients with Meniere's disease and to identify disease-specific proteins, LC-MS/MS was performed with proteins extracted from 1-DE gels from each control and patient. A total of 6784 (C1, FDR of 1.86%), 6108 (C2, FDR of 0.95%), 3114 (C3, FDR of 1.68%), 6961 (P1, FDR of 1.35%), 7740 (P2, FDR of 3.11%), and 763 (P3, FDR of 2.00%) proteins were detected in the LC-MS/MS analysis. Immunoglobulin and its variants were the most commonly identified proteins (49% and 58% of all proteins in the luminal fluid of controls and patients, respectively).

**Figure 3:**
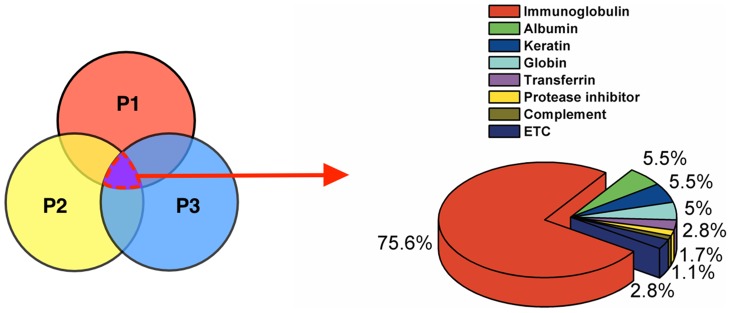
Distribution of common protein components of the endolymphatic sac luminal fluid of Meniere's disease patients. P, patient.

We analyzed the proteins that were found only in the luminal fluid of patients with Meniere's disease. First, common proteins found in the luminal fluid of all of the patients with Meniere's disease were analyzed. A total of 180 proteins were identified in this analysis, 76% of which were immunoglobulin and its variants; albumin, keratin, globin, transferrin, protease inhibitor, and complement were also detected (Fig. 3). Then, proteins that were also detected in controls were excluded from the 180 proteins. As a result, nine proteins were identified: 8 consisted of immunoglobulin and its variants, and 1 was interferon regulatory factor 7 (Table 2 and Table S1). With the exception of the immunoglobulin kappa light chain variable region (gi|4323924), which had a molecular weight of 17–26 kDa, all of the proteins had a molecular weight of 55–63 kDa. Consequently, the proteins detected only in the luminal fluid of Meniere's disease patients were those involved in the inflammatory or immune reactions.

**Table 2:** Proteins identified only in the endolymphatic sac luminal fluid of Meniere's disease patients by LC-MS/MS.

Accession number	Description	Max coverage (%)	Max protein score
gi|34536154	Unnamed protein product	26.3	387
gi|56786126	AF1 non-allergic IgE heavy chain IGHV3-74	27.4	133
gi|1871491	IgM	41.4	180
gi|886286	This CDS feature is included to show the translation of the corresponding V_region. Present translation qualifiers on V_region features are illegal	32.6	102
gi|21668608	Immunoglobulin heavy chain VHDJ region	34.2	69
gi|33319532	Ig heavy chain variable region; VH3 family	52.5	117
gi|33319348	Ig heavy chain variable region; VH3 family	21.7	112
gi|4323924	Immunoglobulin kappa light chain variable region	29	44
gi|1621457	Interferon regulatory factor 7	9.3	64

Protein searches were conducted in MASCOT (version 2.2.04) using the NCBI database. The search parameters were as follows: 1) Enzyme specificity – Trypsin; 2) Maximum missed cleavages –1; 3) Carbamidomethyl (C); Oxidation (M) as variable modifications; MASCOT results were filtered using a protein probability value of less than 0.05. Individual peptide sequences and scores were provided as Table S1.

### Reaction of antigens with patient sera in the Protoarray analysis

**Figure 4:**
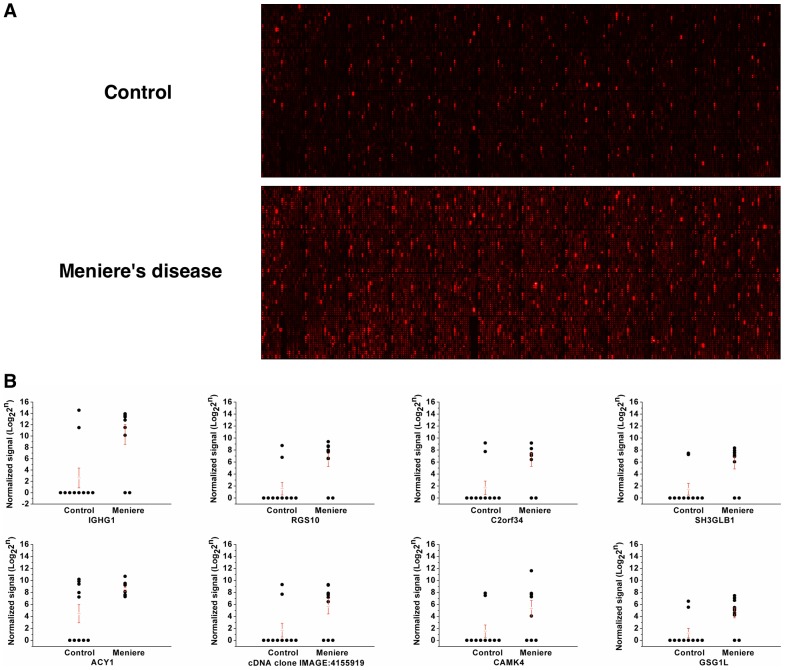
Difference in signal intensity of controls and patients in the Protoarray experiment. A. Raw signals of Protoarray chips of control and Meniere's disease patient. B. Normalized signal intensities of the antigens with a signal intensity more than 10-fold higher in the patients with Meniere's disease than in the controls. IGHG1, immunoglobulin heavy constant gamma 1; RGS10, regulator of G-protein signaling 10, transcript variant 2; C2orf34, chromosome 2 open reading frame 34; SH3GLB1, SH3-domain GRB2-like endophilin B1; ACY1, aminoacylase 1; CAMK4, calcium/calmodulin-dependent protein kinase IV; GSG1L, GSG1-like (GSG1L), transcript variant 2, mRNA. Red bars and error bars represent the mean normalized signal intensity and the SE, respectively.

We investigated the presence of circulating autoantibodies in the peripheral blood of the 10 patients with Meniere's disease using Protoarray Immune Response Biomarker Profiling. Eighteen proteins had more than 2-fold greater signal intensity in the patients with Meniere's disease than in the controls (p<0.05); the signal intensity of 8 proteins was more than 10-fold higher in the patients than in the controls (Table 3, Fig. 4). Among them, the signal intensity of the immunoglobulin heavy constant gamma 1 (IGHG1) was approximately 205-fold higher in the patients than in the controls. A number of antigens had a sensitivity and specificity of >60% and >80%, respectively: IGHG1, the regulator of G-protein signaling 10 (RGS10), transcript variant 2, chromosome 2 open reading frame 34 (C2orf34), and SH3-domain GRB2-like endophilin B1 (SH3GLB1) had a sensitivity and specificity of 80%; the cDNA clone IMAGE:4155919, complete cds, calcium/calmodulin-dependent protein kinase IV (CAMK4), GSG1-like (GSG1L), transcript variant 2, mRNA, and NIMA (never in mitosis gene a)-related kinase 7 (NEK7) had a sensitivity of 70% and a specificity of 80%; and neural cell adhesion molecule 2 (NCAM2) had a sensitivity of 60% and a specificity of 90%. The 8 proteins with more than 10-fold higher signal intensity in the patients had a sensitivity and specificity of >70% and 80%, respectively, for Meniere's disease except Aminoacylase 1 (ACY1) which had a sensitivity and specificity of 100% and 50%, respectively (Fig. 4). More information on antigens with signal intensity more than 2-fold higher in the patients is provided in Table S2. These results provide evidence for the existence of circulating autoantibody and enhanced autoimmune/immune reactions in patients with Meniere's disease.

**Table 3:** Proteins with higher signal intensities in Meniere's disease in the Protoarray analysis.

Accession No.	Swissprot ID	Protein name	Fold change	Function
BC014667.1	P01857	Immunoglobulin heavy constant gamma 1 (G1m marker) (IGHG1)	204.9	Humoral immunity.
NM_002925.3	O43665	Regulator of G-protein signaling 10 (RGS10), transcript variant 2	27.6	Inhibits signal transduction by increasing the GTPase activity of G protein alpha subunits.
BC053733.1	Q7Z624	Chromosome 2 open reading frame 34 (C2orf34)	25.7	Catalyzes the trimethylation of ‘Lys-116’ in calmodulin.
NM_016009.2	Q9Y371	SH3-domain GRB2-like endophilin B1 (SH3GLB1)	20.7	May be required for normal outer mitochondrial membrane dynamics.
NM_000666.1	Q03154	Aminoacylase 1 (ACY1)	16.4	Involved in the hydrolysis of N-acylated or N-acetylated amino acids (except L-aspartate).
BC027465.1	Q8N647	cDNA clone IMAGE:4155919, complete cds	15.8	Unknown.
NM_001744.2	Q16566	Calcium/calmodulin-dependent protein kinase IV (CAMK4)	14.0	Calcium/calmodulin-dependent protein kinase that operates in the calcium-triggered CaMKK-CaMK4 signaling cascade.
NM_144675.1	Q8TB81	GSG1-like (GSG1L), transcript variant 2, mRNA.	10.3	Modifies AMPA receptor (AMPAR) gating.
NM_133494.1	Q8TDX7	NIMA (never in mitosis gene a)- related kinase 7 (NEK7)	9.7	A protein kinase that plays an important role in mitotic cell cycle progression.
NM_003782.3	O96024	UDP-Gal:betaGlcNAc beta 1,3-galactosyltransferase, polypeptide 4 (B3GALT4)	8.1	Involved in GM1/GD1B/GA1 ganglioside biosynthesis.
NM_001011700.1	P59942	Mitochondrial coiled-coil domain protein 1 (MCCD1)	7.5	Unknown.
BC052946.1	O15394	Neural Cell Adhesion Molecule 2 (NCAM2)	6.1	May play important roles in selective fasciculation and zone-to-zone projection of the primary olfactory axons.
NM_000910.1	P49146	Neuropeptide Y Receptor Y2 (NPY2R)	5.9	Receptor for neuropeptide Y and peptide YY.
NM_021644.2	P31942	Heterogeneous Nuclear Ribonucleoprotein H3 (2H9) (HNRPH3), transcript variant 2H9A	3.1	Involved in splicing and participates in early heat shock-induced splicing arrest.
BC050696.1	Q9NWS1	Chromosome 12 Open Reading Frame 48 (C12orf48)	2.8	Plays a central role in DNA repair and in the maintenance of genomic stability by suppressing inappropriate homologous recombination.
BC093864.1	Q92796	Disks large homolog 3 (DLG3)	2.4	Required for learning, most likely due to its role in promoting synaptic plasticity following NMDA receptor signaling.
NM_001157.2	P50995	Annexin A11 (ANXA11), transcript variant	2.0	Required for mid-body formation and completion of the terminal phase of cytokinesis.
BC001304.1	Q32P40	Piccolo (presynaptic cytomatrix protein) (PCLO)	2.0	May be involved in maintaining the neurotransmitter release site in register with postsynaptic reception apparatus.

Protein information was obtained from http://www.uniprot.org/.

Proteins for which the Protoarray signal intensity was more than 2-fold higher in the patients with Meniere's disease than in the controls were listed (p<0.05).

### Reaction of inner ear tissue antigens with patient sera

**Figure 5:**
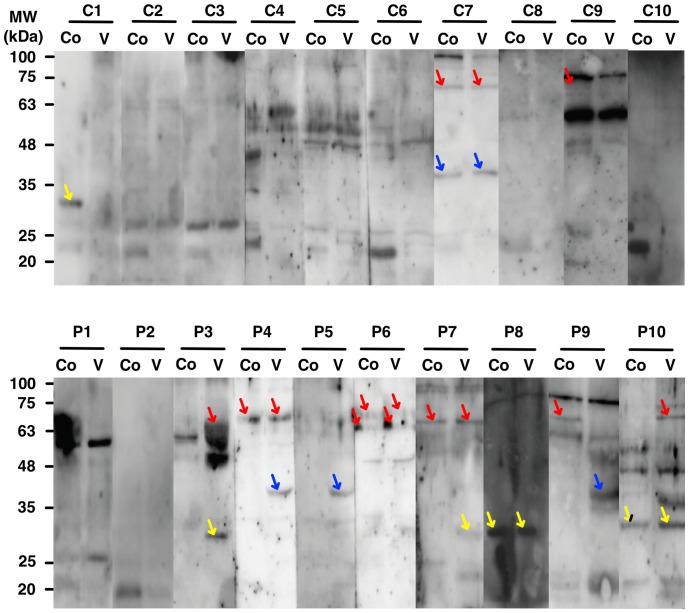
Western blot of the reaction of serum from controls and patients with mouse inner ear. Red, blue, and yellow arrows represent the detected inner ear antigens with molecular weights of 63–75 kDa, 35–48 kDa, and 25–25 kDa, respectively. C, control; P, patient. Co, mouse cochlear tissue protein, V, mouse vestibular tissue protein.

Western blotting (Fig. 5) was performed to investigate whether the sera from patients with Meniere's disease could produce an antigen-antibody reaction with inner ear tissue. There were no single disease-specific bands for an antigen-antibody reaction in the patients; instead bands corresponding to a molecular weight of 63–75 kDa and 25–48 kDa were more frequently found in the patients with Meniere's disease (Fig. 5).

Bands corresponding to a molecular weight of 63–75 kDa were detected in 6 of the patients and in 2 of the controls (red arrows in Fig. 5). Evidence of an antigen-antibody reaction both in the cochlear and vestibular tissues was detected in 3 of the 6 samples from patients with Meniere's disease; evidence of an antigen-antibody reaction only in the vestibular tissues was observed in 2 of the 6 samples, whereas evidence for this reaction only in the cochlear tissues was observed in 1 of the 6 samples. Evidence for an antigen-antibody reaction both in the cochlear and vestibular tissues was observed only in one of the controls (C7) and a weak reaction was observed only in the cochlear tissue in another one of the controls (C9) (Fig. 5).

Bands distributed between 25–48 kDa were detected in 7 of the patients with Meniere's disease and in 2 of the controls (blue and yellow arrows in Fig. 5). The reaction was detected only in the vestibular tissues in 5 of the 7 patient samples and both in the cochlear and vestibular tissues in 2 of the patient samples. The reaction was detected only in the cochlea in 1 of the controls and both in the cochlea and vestibule in the other control.

We evaluated whether the antigens that reacted with patient serum in the Protoarray experiment had molecular weights of 25–48 kDa and 63–75 kDa, as shown in our western blot data. The following proteins had molecular weights in the ranges of interest: C12orf48 (65.1 kDa), PCLO (46 kDa), ACY1 (45.9 kDa), NPY2R (42.7 kDa), B3GALT4 (41.5 kDa), SH3GLB1 (40.8 kDa), HNRPH3, transcript variant 2H9A (36.9 kDa), GSG1L, transcript variant 2, mRNA (36.8 kDa), C2orf34 (36.1 kDa), IGHG1 (36.1 kDa), and NEK7 (34.6 kDa). These antigens could be involved in inner ear autoimmune reactions. Among the proteins, C12orf48 was the only protein which produced a significantly lower signal intensity in the patients without an antigen-antibody reaction in western blots (P1, P2 in Fig. 5) than in the patients with a detectable antigen-antibody reaction in western blots (P3–P10). However, without peptide sequencing, we cannot determine whether the protein was a specific antigen involved in an immune reaction.

Our results imply that multiple autoantibodies or antigens rather than a specific antibody or antigen can cause autoimmune reactions in the inner ear that result in Meniere's disease.

## Discussion

The pathophysiology of Meniere's disease is still unknown, and various etiologies have been proposed. One of the proposed etiologies of Meniere's disease is autoimmunity; this putative etiology is supported by the fact that this disease often occurs bilaterally (in 25–40% of patients), responds to glucocorticoids and anti-inflammatory treatments, and is characterized by elevated levels of autoantibodies or circulating immune complexes and antigen-antibody reactions between patient serum and animal inner ear tissues [18]. However, Meniere's disease does not always occur bilaterally, and experimental studies have only been performed on small numbers of patients or have only targeted a restricted number of autoantibodies or inflammatory markers.

Although the number of patients enrolled in this study was small, we found reliable evidence for the inner ear immune/inflammatory reaction in patients with Meniere's disease by investigating the protein composition of the inner ear fluid in diseased patients and controls. We also mass screened for autoantibody-antigen reactions using the Protoarray system and detected antigen-antibody reactions using patient serum and mouse inner ear tissues. Most of these methods have not been used in previous experiments. The results of this study will contribute to the development of more cost-effective and efficient methods for screening and detecting autoimmune reactions in large numbers of patients with Meniere's disease.

### Analysis of ES luminal fluid and evidences of immune reaction

Autoimmune reactions in the inner ear may cause damage to the epithelial layers surrounding the endolymphatic space. In such an instance, fluid in the endolymphatic space should contain evidence of immunologic reactions, including autoantibodies such as those found in the synovial fluid of patients with rheumatoid arthritis. Thus, analyses of the endolymph can provide evidence for the involvement of autoimmunity in the pathogenesis of Meniere's disease.

We analyzed the protein composition of the inner ear fluid of diseased and control groups to find evidence suggesting that increased immune or inflammatory reaction is involved in the pathogenesis of Meniere's disease. Proteomic techniques (LC-MS/MS) were used to analyze the protein constituents of the inner ear fluid. We used ES luminal fluid because the protein concentrations in this fluid are very high [17] and because the ES is the site where most immunologic reactions occur in the inner ear [19]–[21]. In addition, sampling the ES luminal fluid does not usually affect inner ear function; indeed, the 3 patients enrolled in this experiment who underwent ES surgery had preserved inner ear functions after sampling.

The use of cochlear and vestibular endolymph in addition to ES luminal fluid would have improved our study; however, sampling the cochlear and vestibular endolymph in patients with Meniere's disease would have been impossible because the inner ear function of the patients would have been destroyed and the protein concentrations in these compartments would have been too low for the analysis.

The most commonly encountered proteins in the ES luminal fluid were immunoglobulins and their variants. This is expected, as the ES is a known site of immunologic responses in the inner ear. However, the only proteins that were detected exclusively in the patients with Meniere's disease were immunoglobulins, their variants, and interferon gamma regulatory factor, suggesting that increased inflammatory reactions in the inner ear may contribute to the pathology of Meniere's disease. The increased inflammatory reaction could be caused by various etiologies such as allergy, viral infection, genetic cause, or autoimmunity. Although direct evidence of autoimmunity was not found in the luminal fluid analysis, our results about the presence of circulating autoantibodies and increased immune reaction between patients' sera and mouse inner ear tissue could support the possibility of autoimmunity as a cause of increased immune/inflammatory reaction in the inner ear. If autoimmunity was the cause of the increased inflammatory reactions the autoantibodies responsible for these reactions have a molecular weight between 17 and 26 kDa and between 55 and 63 kDa as revealed by LC-MS/MS in our results.

Evidences for autoimmune reactions in the inner ear of patients with Meniere's disease have been reported. One report demonstrated the presence of focal inflammation with intraepithelial invasion by mononuclear cells in the ES of patients with Meniere's disease that altered the normal structures in the endolymphatic sac [22]. The authors of this study suggested that autoimmune reactions may have triggered the inflammatory changes in the ES.

Another report showed that an antigen-antibody reaction between sera from patients with Meniere's disease and human endolymphatic sac tissue was detected by immunohistochemistry in 10% of patients [23]. However, no study has comprehensively analyzed the protein components of the inner ear fluid of patients with Meniere's disease. This is the first study to investigate the difference in protein constituents of the ES luminal fluid of controls and patients with Meniere's disease and to provide supportive evidence for the involvement of autoimmunity in the pathogenesis of Meniere's disease.

However, there were limitations in our study associated with the use of LC-MS/MS to analyze the ES luminal fluid. First, we could not compare the amounts of certain proteins or peptides, as this technique could only identify the types of proteins contained in the fluid. A comparative analysis with the iTRAQ technique could have quantified the amounts of protein in the controls and patients. However, the amount of ES luminal fluid sampled from each person was too small (less than 200 µl after dilution) to be quantified by iTRAQ and exact quantification after dilution is impossible. In addition, the development of less-invasive therapies such as the use of intratympanic steroids or gentamicin injections has limited the need for ES surgery and therefore the opportunities to sample ES luminal fluid. Additionally, although many immunoglobulins and their variants were detected in the LC-MS/MS analysis, it remains unclear whether these proteins acted as autoantibodies. Immunoprecipitation using human inner ear fluid and the inner ear or using human inner ear fluid and animal inner ear samples would need to be performed, and the identity of the autoantibody would need to be confirmed with mass spectrometry. However, these experiments would be difficult to perform for ethical reasons and because of the limited amounts of human inner ear fluid. Microanalysis techniques with very small amounts of sample should be performed to confirm the occurrence of autoimmunity in the future.

### Evidence of circulating autoantibodies in patient sera

We sought to determine whether circulating autoantibodies were present in the sera of the patients, as inner ear fluid is difficult to sample for practical use; this limitation precludes the study of the pathologic mechanisms underlying Meniere's disease in large numbers of patients. Detectable autoantibodies or evidence of autoimmunity in the serum could be used as diagnostic biomarkers in conjunction with mass screening. There were several reports investigating the difference of serum protein profiles between Meniere's disease patients and controls using proteomics as a screening method. Proteins related to immune reaction or inner ear disorder such as complement factor H, β2 glycoprotein 1, vitamin D binding protein, and β actin were previously reported to be increased in the serum of Meniere's disease by proteomic analysis [15]. Additionally, fibrinogen α and γ chains, β2 glycoprotein, and complement factor B and H were revealed to be differently expressed according to hearing threshold of patients [24]. However, this study eliminate serum abundant proteins such as albumin and immunoglobulins for 2-DE analysis and performed LC-MS/MS in the several spots that were different between Meniere's disease and control after image analysis. Therefore, most circulating antibodies could be excluded in the analysis and only targeted spots were analyzed by LC-MS/MS; the study did not focused on the Ag-Ab reaction between circulating antibodies and corresponding antigens, but analyzed proteins exclusively increased in the serum of Meniere's disease patients. In our study, we tried to investigate Ag-Ab reaction between circulating antibodies and target antigens using Protoarray and consequently detected circulating antibodies to 18 candidate proteins which could be involved in autoimmune reactions in the patients; the signal intensity of these proteins was more than 2-fold higher in the patients with Meniere's disease than in the controls. With the exception of IGHG1, all of these proteins were located in the membrane or subcellular areas (information on the localization of these proteins can be found at http://www.uniprot.org). These proteins are known to be involved in cell signaling, mitochondrial structure, receptor gating, cell mitotic activity, ganglioside biosynthesis, fasciculation, neuropeptides, DNA splicing and repair, cytokinesis, and maintaining neurotransmitter release sites (Table 3). However, the functions of some of these proteins, including cDNA clone IMAGE:4155919, complete cds, and Mitochondrial coiled-coil domain protein 1 (MCCD1), are unknown. Among those proteins, only mRNA of CAMK4, NEK 7, and PCLO were reported to exist in the inner ear sensory epithelium and ribbon synapse [25], but the existence of the other proteins in the human inner ear has not been demonstrated. PCLO is thought to be involved in the synaptic neurotransmission in the inner ear, although its functional relevance is still unclear [26]. If autoantibodies for PCLO in Meniere's disease deteriorate the inner ear function by reacting with PCLO in the ribbon synapse, it could be one of the candidate autoantibodies for the disease. However, a truncated splice variant of PCLO, piccolino, which could maintain the integrity of synaptic transmission in the absence of PCLO in the retina ribbon synapse, was also found in the sensory ribbon synapse of the cochlea [27]. Therefore, the synaptic transmission can remain intact, even with the Ag-Ab reaction between autoantibodies and PCLO in the inner ear. The function of the other proteins which were reported to exist in the inner ear still remains unknown.

The enhanced immune reaction associated with IGHG1 could be explained in several ways. First, an immune complex autoimmune disorder could explain this occurrence. Circulating autoantibodies to immunoglobulins could form immune complexes that may cause inner ear damage via a type III hypersensitivity reaction. Indeed, several studies have reported elevated circulating immune complexes in 54–94% of patients with Meniere's disease [28], [29]. However, other studies have shown evidence of circulating immune complexes in only 4–7.4% of patients [30], [31]. Several methods can be used to detect circulating immune complexes; however, none of these methods can detect all types of circulating immune complexes. Therefore, the prevalence of patients with circulating immune complexes may vary from study to study. Differences in the race and number of patients enrolled in a study can also influence the prevalence of circulating immune complexes. Evidence of damage to the inner ear associated with circulating immune complexes is important in the pathogenesis of Meniere's disease. However, evidence for inner ear pathology is insufficient, even though histopathological studies of the human temporal bone have demonstrated that patients with Meniere's disease have C3 and C1q deposits in their inner ear [32], [33].

Second, increased amounts of anti-IGHG1 antibodies in patients with Meniere's disease may be a result of an excessive autoimmune or inflammatory reaction in the inner ear. It is also possible that increased concentrations of anti-IGHG1 antibody are involved in regulating the immune system and suppressing excessive immune reactions by reducing B cell activity. Other antibodies may cause autoimmune reactions at a cellular level and affect the function of the epithelial cells of the inner ear that regulate inner ear homeostasis via the disruption of cell signaling or cellular structures. Such autoimmune reactions could result in secondary increases in the concentrations of anti-immunoglobulin antibodies.

### Evidence of an immune reaction between patient serum and the inner ear

Demonstrating the presence of immune reaction between circulating autoantibodies in the serum and the inner ear tissue is important. We used western blots with proteins from mouse inner ear tissue and patient serum to demonstrate the existence of this type of immune reaction. The western blots showed that more antigen-antibody reactions occurred in the patients with Meniere's disease than in the controls. Animal inner ear antigens that have been reported to react with sera from patients with Meniere's disease have molecular weights in the 32–35 kDa, 42–46 kDa, 52–59 kDa, and 79–80 kDa ranges [18]. Microsequencing showed that the 28 KDa and 42 KDa antigens corresponded to Raf-1 and beta actin, respectively [18]. These antigens have been detected in a variety of species, including guinea pigs, cows, and humans, suggesting that the antigens might be organ-specific rather than species-specific. In our experiment, inner ear antigens with molecular weights in the 25–35 kDa, 35–48 kDa, and 57–63 kDa ranges were detected. These antigens are likely to be similar to those detected in previous studies. Although the identity of these antigens can be conjectured from the antigens with similar molecular weight detected in the Protoarray experiment as suggested in the result, it should be further studied using immunoprecipitation of patient serum and inner ear tissues followed by mass spectrometry of the corresponding protein bands.

We divided the mouse inner ear tissue into cochlear and vestibular tissues and investigated whether an antigen-antibody reaction between these tissues and patient serum occurred. In contrast, previous studies tended to use whole inner ear tissue. We found that each antigen reacted with the serum differently; samples of patient serum could react with the cochlear tissue, with the vestibular tissue, or with both. Clinically, cochlear and vestibular symptoms in Meniere's disease are different for each patient. In general, vestibular symptoms tend to coincide with cochlear symptoms. However, the progression of each cochlear and vestibular symptom and function varies from patient to patient. The varying antigen-antibody reactions observed in each tissue may be associated with the varying clinical features of the disease.

Because a variety of inner ear antigens could react with patient serum, it appears that multiple target antigens and autoantibodies (rather than a single antigen-antibody combination) may be responsible for the autoimmune reaction associated with Meniere's disease. The 1-DE findings examining the protein composition of the ES luminal fluid of patients with Meniere's disease also support this hypothesis: the distribution of bands was different in the 3 patients, suggesting that the protein composition of the ES luminal fluid of each patient was different and that different antibodies or inflammatory materials are present in each patient.

### Clinical and future implications

The diagnosis of Meniere's disease is based on clinical symptoms of vertigo and fluctuations in hearing; the diagnosis is confirmed by showing evidence of sensorineural hearing loss using the pure tone audiogram, as recommended by the AAO-HNS [1995] [16]. Other clinical tests, including vestibular function tests, electrocochleography, and the glycerol test, are not as useful for diagnosing Meniere's disease. Because the diagnostic criteria are primarily symptom-based, differentiating Meniere's disease from other vestibular disorders such as vestibular migraine, vestibular paroxysmia, and sudden sensorineural hearing loss with vertigo can be difficult. Diagnostic markers that can more accurately diagnose Meniere's disease are therefore needed. Biologic markers can potentially decrease the cost of diagnosing Meniere's disease by avoiding unnecessary laboratory and imaging work-ups and promoting proper treatment after accurate diagnosis. If autoimmunity is one of the causes of Meniere's disease, detecting autoantibodies or inflammatory materials can be useful.

Highly sensitive experimental chips using candidate antigens (such as the 18 antigens detected in our study) can be manufactured to avoid the need to use the expensive conventional screening chips used in our study. These diagnostic chips should contain multiple candidate antigens, as multiple candidate antigens were detected in our study and in previous reports. A prospective study using experimental chips should be undertaken in a large population, and highly sensitive and specific markers should be chosen. This type of study will enable subtypes and the prognosis of patients with Meniere's disease to be classified. Understanding the pathophysiology underlying Meniere's disease can also contribute to the development of new treatment methods. None of the current treatments for Meniere's disease can prevent the progression of the disease. Prospective, randomized controlled studies using anti-inflammatory agents such as TNF-α or steroids would be performed in a large population in the patients with autoimmune pathology if it can be confirmed by the autoimmunity screening.

Many studies have attempted to describe the pathophysiology of Meniere's disease; however, our understanding of the pathophysiology of this disease remains limited. In fact, Meniere's disease is a syndrome which may be caused by multiple factors. Autoimmunity is one of the candidate etiologies and thought to represent less than 20%. Recently, familial aggregation for Meniere's disease was reported to be as high as 10–20% [6], [8]. This represents a significant role for genetics in Meniere's disease. Among the various candidate genes associated with Meniere's disease, several genes related to immune response were reported to determine an increased susceptibility of Meniere's disease. Genes that were revealed to be associated with bilateral Meniere's disease, chronic balance/hearing loss were allelic variants of DRB1, PTPN22, TLR 10, MICA genes [34]–[36]. Genetic factors can be one of the causes of autoimmunity or increased immune reaction in Meniere's disease. If various candidate genes associated with Meniere's disease are revealed in the future, it can also be used as evidence for developing new treatment method as well as diagnostic and prognostic markers. However, so far, at least 60–70% of etiologies of Meniere's disease remains unknown, and considerable efforts should be taken to investigate the etiopathogenesis of Meniere's disease using new molecular technologies.

## Conclusion

The findings of this study suggest that autoimmunity could be one of the pathologic mechanisms behind Meniere's disease. Multiple autoantibodies and antigens may be involved in the autoimmune reaction. Specific antigens that caused immune reactions with patient's serum in Protoarray analysis can be candidates for the diagnostic biomarkers of Meniere's disease. Further studies with mass screening using candidate antigen-antibody reactions are needed to identify future treatment modalities and to determine the true prevalence of autoimmune pathologic mechanisms underlying Meniere's disease.

## Supporting Information

Table S1 LC-MS/MS profiles of the 8 proteins that were found only in the 3 patients with Meniere's disease. Each sheet of Excel data presents the protein profile of each patient.
(XLS)

Table S2 Signal intensities of antigen-antibody reactions and peptide sequences of the 18 antigens with a Protoarray signal intensity more than 2-fold higher in the patients than in the controls.
(XLS)
